# Valorization of Hemp Hurds as Bio-Sourced Additives in PLA-Based Biocomposites

**DOI:** 10.3390/polym13213786

**Published:** 2021-11-01

**Authors:** Sina Momeni, Muhammad Safder, Mohammad Abu Hasan Khondoker, Anastasia Leila Elias

**Affiliations:** 1 Donadeo Innovation Centre for Engineering, Department of Chemical and Materials Engineering, University of Alberta, Edmonton, AB T6G 1H9, Canada; smomeni@ualberta.ca (S.M.); safder@ualberta.ca (M.S.); Mohammad.Khondoker@uregina.ca (M.A.H.K.); 2 Industrial Systems Engineering, Faculty of Engineering and Applied Science, University of Regina, Regina, SK S4S 0A2, Canada

**Keywords:** hemp hurd powder, alkaline treatment, alkaline/peroxide treatment, filler-matrix interaction

## Abstract

Sourced from agricultural waste, hemp hurds are a low-cost renewable material with high stiffness; however, despite their potential to be used as low-cost filler in natural fiber reinforced polymer biocomposites, they are often discarded. In this study, the potential to add value to hemp hurds by incorporating them into poly(lactic acid) (PLA) biopolymer to form bio-based materials for packaging applications is investigated. However, as with many plant fibers, the inherent hydrophilicity of hemp hurds leads to inferior filler-matrix interfacial interactions, compromising the mechanical properties of the resulting biocomposites. In this study, two chemical treatments, alkaline (NaOH) and alkaline/peroxide (NaOH/H_2_O_2_) were employed to treat hemp hurds to improve their miscibility with poly(lactic acid) (PLA) for the formation of biocomposites. The effects of reinforcement content (5, 10, and 15 wt. %), chemical treatments (purely alkaline vs. alkaline/peroxide) and treatment cycles (1 and 3 cycles) on the mechanical and thermal properties of the biocomposites were investigated. The biocomposites of treated hemp hurd powder exhibited enhanced thermal stability in the temperature range commonly used to process PLA (130–180 °C). The biocomposites containing 15 wt. % hemp hurd powder prepared using a single-cycle alkaline/peroxide treatment (PLA/15APHH1) exhibited a Young’s modulus of 2674 MPa, which is 70% higher than that of neat PLA and 9.3% higher than that of biocomposites comprised of PLA containing the same wt. % of untreated hemp hurd powder (PLA/15UHH). Furthermore, the tensile strength of the PLA/15APHH1 biocomposite was found to be 62.6 MPa, which was 6.5% lower than that of neat PLA and 23% higher than that of the PLA/15UHH sample. The results suggest that the fabricated PLA/hemp hurd powder biocomposites have great potential to be utilized in green and sustainable packaging applications.

## 1. Introduction

Environmental issues associated with both plastic waste and depletion of petroleum resources have been a major driving force for the production and utilization of biodegradable biopolymers. Among biodegradable biopolymers, poly(lactic acid) (PLA) is attractive for a variety of applications owing to its biocompatibility [1], renewability [2], and promising mechanical properties [3,4]. Despite these key advantages, PLA has had only limited adoption in fields such as packaging, the automotive industry, and construction, mainly because of some crucial drawbacks including brittleness, low thermal stability, and relatively high production cost. Incorporation of reinforcing agents, plasticizers, and compatibilizers are among the proposed strategies to tailor or enhance the properties of PLA [5,6,7,8,9].

In recent years, natural fiber-reinforced biopolymers have received extensive interest due to the “green” nature of these biocomposites. Moreover, natural fibers have positive characteristics including desirable mechanical properties, low density, and low cost, which make them a promising candidate as reinforcing fillers [10,11]. Among reinforcing agents, hemp fiber accounts for one of the most extensively studied natural fibers in the literature. Derived from the hemp plant, hemp fiber (also called bast fiber) is rich in cellulose (70–74%) and is known as one of the stiffest and strongest natural fibers available for industrial use, and thus is considered as a desirable reinforcing material [11]. Bast fibers, which grow on the outer core of the hemp stalk, can be meters in length, and are one of the most highly valued parts of the plant. The hemp plant additionally includes hurds, the lightweight soft woody core of the hemp plant, which are often considered to be waste, and are therefore lower in cost than bast fibers. Hemp hurds also have a high cellulose content (40–48%), and therefore cellulose extracted from hurd is still a good candidate for use as a reinforcing agent in polymers [12,13]. However, like other natural fibers, hemp hurds also contain non-cellulosic components such as hemicellulose, lignin, pectin, and wax; these components tend to have low thermal stability and high hydrophilicity, which may result in poor filler-matrix interfacial interaction as well as inferior processibility during the formation of biocomposites [14,15,16]. To overcome these challenges, surface modification of natural fibers through chemical treatments (e.g., alkalization [17], silanization [18], acetylation [19]), physical treatments (e.g., plasma [20,21] and thermal steam explosion [22]), and biological treatments (e.g., enzyme [23]) are typically performed before biocomposite processing.

Among surface modification techniques, chemical treatments of natural fibers have been studied extensively in the past few years. Alkaline treatment is a common chemical treatment to remove non-cellulosic constituents from natural fibers due to its simplicity and low cost [14,24]. Numerous studies have reported superior mechanical and thermal properties of composites containing fibers pretreated with alkaline solutions as compared with composites containing untreated fibers [25,26,27]. For instance, Sawpan et al. observed that the incorporation of hemp fibers treated with 5 wt. % NaOH solution in a PLA matrix increased the Young’s modulus, tensile strength, and impact strength of biocomposites as compared with composites containing either untreated or silane-treated fibers [28]. Sawpan et al. inferred that the aforementioned improvements could be attributed to a strong fiber-matrix interfacial interaction caused by the removal of hemicellulose and impurities from the fiber surface after alkaline treatment. However, it should be noted that higher concentrations of NaOH might result in the degradation of fibers which can adversely affect the mechanical and thermal properties of the reinforced composites. Pickering et al. evaluated the impact of alkaline treatment on the mechanical properties of hemp fibers [29]. Their results indicated that using 15 wt. % NaOH solution for treatment purposes adversely affected the strength of fibers as a consequence of excessive delignification and degradation of cellulose.

Bleaching with hydrogen peroxide (H_2_O_2_) is an alternative chemical treatment that is extensively used in the textile industry [30]. It has been reported that hydrogen peroxide treatment can improve both the physical appearance and mechanical properties of fibers due to the partial removal of lignin [31]. To increase the efficiency of bleaching, it is crucial to perform the hydrogen peroxide treatment in an alkaline medium [32]. Zhang et al. evaluated the effect of alkaline/peroxide treatment on the properties of citrus dietary fibers by dispersing the fibers in 1% hydrogen peroxide solution with 0.5 M NaOH solution. They observed that after alkaline/peroxide treatment, lignin and pectin contents were reduced and the treated fibers were found to be richer in cellulose [33]. In another study, Razak et al. investigated the impact of alkaline/peroxide treatment (5% *v*/*v*) on the mechanical properties of PLA/kenaf biocomposites. They reported that the biocomposites formed with the bleached fibers showed higher tensile strength, Young’s modulus, and elongation at break compared to biocomposites containing unbleached fibers [31]. However, the effect of multiple cycles of treatment was not investigated by any of these studies. 

One obstacle to the widespread use of PLA in packaging and other single-use applications is cost; for instance, the bulk cost of PLA is more than USD 2/kg as compared to <USD 0.5 /kg for polyethylene (PE). If the cost of this polymer can be reduced through the incorporation of an inexpensive biodegradable filler without compromising the properties of the material overall, PLA may find more use in applications such as packaging. Most of the studies in the literature used bast fibers (valuable and relatively expensive part of plants that are also used in applications such as textiles) as reinforcing agents for PLA-based biocomposites. While the use of these reinforcing agents can often result in improvements in the thermal and/or mechanical properties of PLA-based composites, due to the high cost of the fibers themselves (which are generally more expensive per kg than PLA resin), the resulting composites may have limited use. Very few studies have reported the use of hemp hurds—an underutilized part of the hemp plant that is often considered a by-product [34]—as reinforcing agents in biopolymers. Incorporation of hemp hurds in PLA matrix may not only reduce the overall cost of the final product—due to the very low cost of hemp hurds—but also help to find a sustainable application for this material, which is often considered as agricultural waste. 

In this work, we prepared PLA/hemp hurd powder biocomposites, and characterized their thermal and mechanical properties as a function of hemp hurd powder treatment and concentration to: (1) add value to hemp hurds by using them as low-cost filler, (2) find suitable end-of-life application for by-products of the hemp industry, (3) develop bio-based and potentially biodegradable material that can be used in commercial applications. Hemp hurd powder was prepared by grinding hemp hurds to obtain a powder with a size of less than 500 microns. The hemp hurd powder was subjected to two different types of chemical treatments (alkaline and alkaline/peroxide), which were selected to achieve good interactions between the inherently hydrophilic hemp hurds [35] and the hydrophobic PLA [2] matrix. These treatments were expected to remove the undesirable components (i.e., hemicellulose, lignin, pectin, and wax) of the hemp hurds, thereby increasing the cellulose fraction of the material. While increasing the number of treatment cycles may reduce the fraction of undesirable components and enhance the fraction of cellulose in the resulting material, too many cycles may actually damage the hemp hurds. We therefore examined how the properties of both hemp hurds and of PLA/hemp hurd powder biocomposites were impacted by repeating multiple cycles of these treatment processes. Overall, the effects of the type of chemical treatment, number of treatment cycles, and hemp hurd powder content on the mechanical and thermal properties of the fabricated PLA/hemp hurd powder biocomposites were investigated, toward developing a material that can be used in the packaging industry, especially in applications which require relatively stiff materials.

## 2. Materials and Methods

### 2.1. Materials

Poly(lactic acid) (PLA, nominal Mw: 390 kDa, grade 4043D pellets) was purchased from NatureWorks LLC, Minnetonka, MN, USA. Hemp hurds were provided by the Vegreville Decortication Facility, Vegreville, AB, Canada. Required chemicals such as sodium hydroxide (NaOH) pellets (relative density: 2.13 g/cm^3^ at 20 °C, purity: ≥98%) and hydrogen peroxide (H_2_O_2_) solution (30 wt. %, relative density: 1.11 g/cm^3^) were purchased from Sigma Aldrich^®^, Oakville, ON, Canada, and used as received without any processing.

### 2.2. Preparation of Hemp Hurd Powders

The visible dirt of the received hemp hurds was removed by vigorous washing with 60 °C water for 2 h. After drying at 90 °C for 24 h, washed hemp hurds were ground using a blade coffee grinder (Model 80392, Hamilton Beach^®^, Glen Allen, VA, USA) for 3 min; the resulting powder was sieved using a 500 µm size screen (35 mesh, Fieldmaster^®^, Science First LLC, Yulee, FL, USA). This material was denoted as untreated hemp hurd powder (UHH). Once prepared, the ground hurds were stored in sealed plastic bags at room temperature until their use.

#### 2.2.1. Alkaline Treatment

The alkaline treatment started with sonicating 5 g of ground hemp hurds in 300 mL of distilled water for 30 min in an ultrasonic bath (Model 15337401, Fisher Scientific, Hampton, NH, USA) to dissolve water-soluble hemicellulose. Afterward, the mixture was vacuum filtered using a 20 µm size screen. The filtered wet powder was then put in 100 mL of 8 wt. % NaOH solution and sonicated at room temperature for 1 h. The solution was then kept at room temperature (without sonication) for 47 h. Then, the sample was vacuum filtered using a 20 µm size screen and washed with distilled water containing 1% acetic acid to neutralize the remaining caustic soda, and then thoroughly washed with distilled water until a pH of ~7 was reached. Finally, the hurd powder was dried at 90 °C for 24 h. The resulting powder was denoted as single-cycle alkaline-treated hemp hurd powder (AHH1). The same treatment procedure was repeated twice more to obtain three-cycle alkaline-treated hemp hurd powder (AHH3).

#### 2.2.2. Alkaline/Peroxide Treatment

After washing and vacuum-filtering of 5 g of hemp hurd powder using the procedure explained in the alkaline treatment Section 2.2.1, the wet powder was added to 100 mL of 8 wt. % NaOH solution and sonicated at room temperature for 10 min. Then, 100 mL of 11% *v*/*v* hydrogen peroxide solution was added in the mixture, which was vigorously stirred for 90 min at room temperature. This treatment process was inspired by Zhang et al., who observed that performing alkaline hydrogen peroxide treatment improved the properties of citrus dietary fiber [33]. The sample was then filtered and washed with distilled water until a pH of ~7 was reached. Finally, the powder was dried at 90 °C for 24 h. The complete cycle includes both alkaline and hydrogen peroxide treatments, and the obtained powder is referred to as single-cycle alkaline/peroxide-treated hemp hurd powder (APHH1). In some cases, the same two-part treatment procedure was repeated twice more to prepare three-cycle alkaline/peroxide-treated hemp hurd powder (APHH3). The images corresponding to untreated and treated hemp hurd powders are shown in the Supplementary Information, Figure S1.

### 2.3. Biocomposite Fabrication

Before biocomposite fabrication, hemp hurd powder and PLA were dried at 70 °C in an oven overnight to minimize the moisture content. The compounding was carried out in a co-rotating twin-screw extruder (HAAKE™ Minilab II micro compounder, Thermo Fisher Scientific, Waltham, MA, USA) at 160 °C with a rotor speed of 75 rpm. All batches were cycled through the extruder channel for 3 min (the residence time) to ensure proper melt-mixing and then extruded through a rectangular nozzle. The extruded filaments were cooled to room temperature and then pelletized manually using scissors. The resulting pellets were rectangular in shape, typically 2–3 mm in length, 2 mm in width, and 0.5–1 mm in thickness. As per the ASTM D638-14 test [36], standard Type I test specimens were prepared using a hot-pressing machine (Model 4386, Carver Inc., Wabash, IN, USA) and a rectangular steel mold 2 mm thick with dimensions of 70 mm × 80 mm. Samples were maintained under 2 MPa pressure at 180 °C for 5 min (these parameters were selected to minimize the number of bubbles that formed in the samples). Once cooled down to room temperature, dumbbell-shaped specimens were removed out of the mold and stored in a sealed bag before tensile testing. The schematic of the dumbbell-shaped specimens is shown in the Supplementary Information, Figure S2.

Throughout this work, each biocomposite is referred to in the format of “PLA/filler type”, where the number before the filler type refers to hemp hurd powder content; for instance, PLA/15APHH3 denotes PLA/hemp hurd powder biocomposites containing 15 wt. % of three-cycle alkaline/peroxide-treated hemp hurd powder. The designated names for hemp hurd powders and prepared biocomposites are summarized in Table 1.

### 2.4. Characterization

#### 2.4.1. Fourier-Transform Infrared Spectroscopy (FTIR)

The chemical analysis of untreated and treated hemp hurd powders was performed using an FTIR spectrometer (MB3000, ABB Inc., Zurich, Switzerland) equipped with ATR (attenuated total reflectance) to determine the interfacial bonds. Each spectrum was obtained in the spectral range of 4000 to 400 cm^−1^ with a scanning resolution of 4 cm^−1^. A total number of 120 scans were taken for each sample and averaged. 

#### 2.4.2. Scanning Electron Microscopy (SEM)

The morphological analysis of untreated and treated hemp hurd powders was carried out using SEM analysis (Model ZEISS EVO MA 10, Jena, Germany). Samples were placed on aluminum holders with double-sided conductive tape. Prior to carrying out SEM characterization, a Denton sputter unit was used to coat the samples with a thin layer of gold.

#### 2.4.3. X-ray Diffraction (XRD) Analysis

The X-ray powder diffraction analysis of untreated and treated hemp hurd powders was performed using an X-ray diffractometer (Bruker XRD D8 Discover, Billerica, MA, USA). The diffracted intensity was recorded at a scanning rate of 0.5°/min between 5 and 40° diffraction angle at 40 kV and 30 mA using locked couple mode. The crystallinity index (*I_c_*) was calculated using the Segal method [37], a well-established method for calculating the crystallinity of cellulosic materials. This technique relies on comparing the intensity of two features: (1) the diffraction peak located at ~22.5° that corresponds to the (002) plane (crystalline region) of cellulose, and (2) a local minimum at around 18° corresponding to the less ordered components of the cellulose (often denoted as amorphous) [38,39]. The crystallinity index is calculated from the intensity of the (002) peak (*I*_002_) and the intensity observed at the local minimum at ~18° (*I_am_*) using Equation (1):

(1) $$I_{c}\;(\%)=\frac{I_{002}-I_{am}}{I_{002}}\;\times \;100$$

#### 2.4.4. Thermo-Gravimetric Analysis (TGA)

The thermal stability of the hemp hurd powders and biocomposites was investigated using TGA (TGA/DSC Model 1, Mettler Toledo, Columbus, OH, USA) equipped with an ultra-micro balance cell and differential thermal analysis (DTA) sensors. An approximately 10 mg sample was placed in an alumina crucible and heated from 25 to 799 °C at a heating rate of 10 °C/min under a nitrogen atmosphere with a flow rate of 20 mL/min.

#### 2.4.5. Differential Scanning Calorimetry (DSC)

Thermal analysis and crystallization behavior of PLA and the biocomposites were investigated by use of a differential scanning calorimeter (DSC Model 1, Mettler Toledo, Columbus, OH, USA). Samples with a mass of approximately 5 mg were placed in sealed aluminum crucibles and then heated from −20 to 200 °C at a heating rate of 5 °C/min. Following the first heating scan, samples were held at 200 °C for 5 min and then cooled down to −20 °C at a cooling rate of 5 °C/min. Finally, samples were heated again to 200 °C at a heating rate of 5 °C/min to obtain the second heating cycle behavior. The degree of crystallinity (*X_c_*) of PLA and its biocomposites were calculated using Equation (2):

(2) $$X_{c}\;(\%)=\frac{\Delta H_{m}}{w\;\times \;\Delta H_{m}^{0}}\;\times \;100$$

where *w* is the weight fraction of PLA in the biocomposites, Δ*H_m_* corresponds to the melting enthalpy of biocomposite, and $\Delta H_{m}^{0}$ refers to melting enthalpy of pure crystalline PLA (96 J/g) [9].

### 2.5. Mechanical Testing of Biocomposites

Tensile properties of PLA and the biocomposites were evaluated based on the Type V test coupon of ASTM D638−14 standard, using a universal testing machine Instron 5943 (Instron, Norwood, MA, USA) equipped with a 1 kN load cell at a crosshead speed of 5 mm/min. At least 5 samples were measured for each composite type and the average value is reported. As shown in Figure S2, overall length (LO), distance between grips (D), gauge length (G), width of narrow section (W), and thickness (T) of the dumbbell-shaped specimens were 75, 25, 7.62, 4, and 2 mm, respectively. Fabricated dumbbell-shaped specimens after tensile testing are shown in the Supplementary Information, Figure S3.

## 3. Results and Discussion

### 3.1. FTIR Analysis

**Figure 1:**
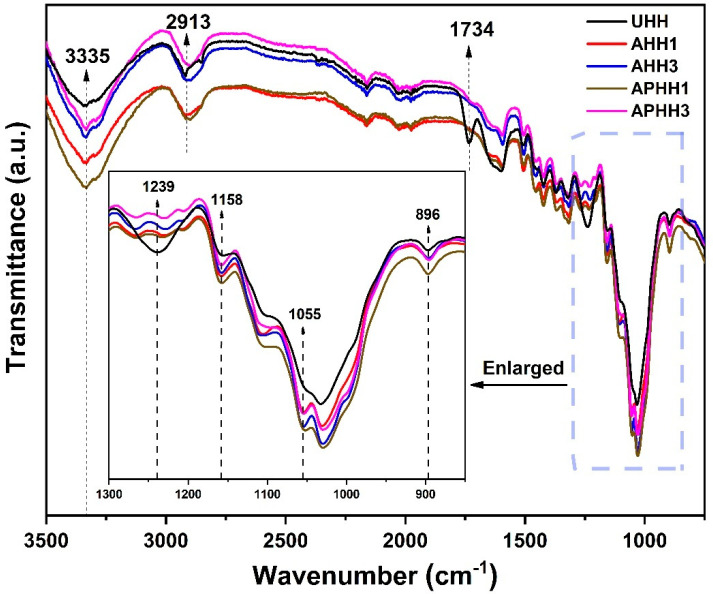
FTIR spectra of untreated and treated hemp hurd powders.

FTIR spectra of untreated, alkaline, and alkaline/peroxide treated hemp hurd powders are shown in Figure 1. The characteristic peaks are located at the following wavenumbers: 3335 cm^−1^ (OH stretching in cellulose, hemicellulose) [16], 2913 cm^−1^ (CH stretching in cellulose) [15], 1734 cm^−1^ (C=O stretching of acetyl groups in hemicellulose) [15,40], 1239 cm^−1^ (C-O stretching of acetyl groups in lignin) [40,41,42], 1158 cm^−1^ (asymmetrical C-O-C stretching in cellulose, hemicellulose) [43], 1100–1000 cm^−1^ (C-O and C-O-C stretching vibrations of cellulose) [40,42,44,45], 896 cm^−1^ (symmetrical β-glycoside bonds ring-stretching in cellulose) [43]. For all of the hemp hurd powders (both treated and untreated), the characteristic peaks of cellulose at 3335, 2913, and 896 cm^−1^ can be clearly observed in Figure 1.

The main changes in the spectra observed after alkaline and alkaline/peroxide treatment were the notable decrease in the intensity of the peaks at 1734 and 1239 cm^−1^ corresponding to hemicellulose and lignin, respectively, suggesting the removal of these groups. The vanishing of these peaks has been noted previously in other studies: Kathirselvam et al. [46] noted that the band at 1734 cm^−1^ vanished after NaOH treatment of cellulose fibers from Thespesia populnea bark, and attributed this change to the removal of hemicellulose. Viscusi et al. [42] observed a similar disappearance at 1732 cm^−1^ after ball mill + NaOH treatment of hemp fibers, and attributed the change to the removal of both hemicellulose and lignin. Oza et al. [14] studied the effects of alkaline (and other treatments) on the properties of industrial hemp fibers; they noted the vanishing of a broader peak from 1740 to 1750 cm^−1^, attributed to the removal of wax and pectin. In studies [14,42], similar vanishing of peaks at 1250 and 1247 cm^−1^ were observed after NaOH treatment, corresponding to the removal of lignin. Overall, the changes in the spectra of the treated hemp hurd powder show that lignin and hemicellulose were at least partially removed during the chemical treatment. 

### 3.2. SEM Analysis

**Figure 2:**
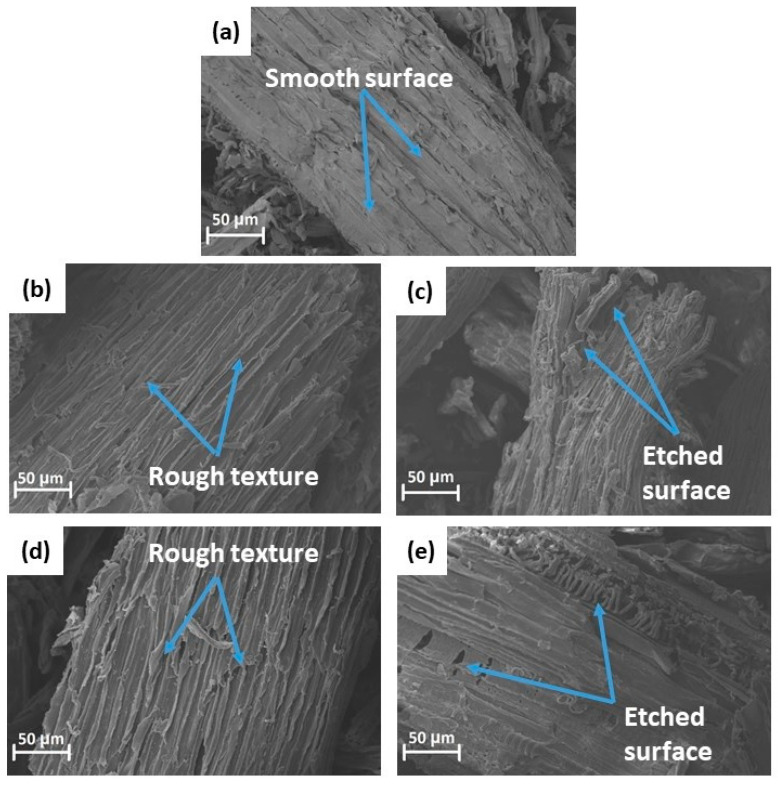
SEM micrographs of untreated hemp hurd powder (**a**), treated hemp hurd powder exposed to one and three cycles of alkaline treatment ((**b**,**c**) respectively), and hemp hurd powder exposed to one and three cycles of alkaline/peroxide treatment ((**d**,**e)** respectively).

The effect of alkaline and alkaline/peroxide treatments on the morphology of the hemp hurd powder can be seen by comparing the SEM images of untreated and treated hemp hurd powders shown in Figure 2. The impurities covering the surface of untreated hemp hurd powder (Figure 2a), including wax, hemicellulose, and lignin, account for the smooth surface. For hemp hurd powder exposed to chemical treatment, a rougher surface texture is visible, which can be attributed to the fibrillation during the treatment process. Interestingly, the degree of surface roughness of the hemp hurd powder increased when performing multiple treatment cycles. For instance, Figure 2b,d shows the mild surface roughness of hemp hurd powder after only one cycle of treatment. In contrast, Figure 2c,e shows higher surface roughness of hemp hurd powder resulting from three-cycle alkaline and alkaline/peroxide treatments, respectively. The increasing roughness visible with increasing number of treatment cycles suggests that either (1) the hemicellulose and lignin were being removed more completely by multiple cycles, or (2) that the cellulose itself may have been damaged by increasing number of treatments.

The results of the alkaline treatment (which used an 8 wt. % NaOH solution) are in agreement with the literature. Kathirselvam et al. treated raw cellulose fibers with 2, 5, and 8 wt. % of NaOH solution for 60 min at room temperature [46]. They observed that while a mild concentration (2 wt. %) of alkaline solution did not noticeably alter the surface of the fibers, fibers treated with 5 and 8 wt. % had a rougher surface attributable to the complete removal of wax and impurities.

### 3.3. XRD

**Figure 3:**
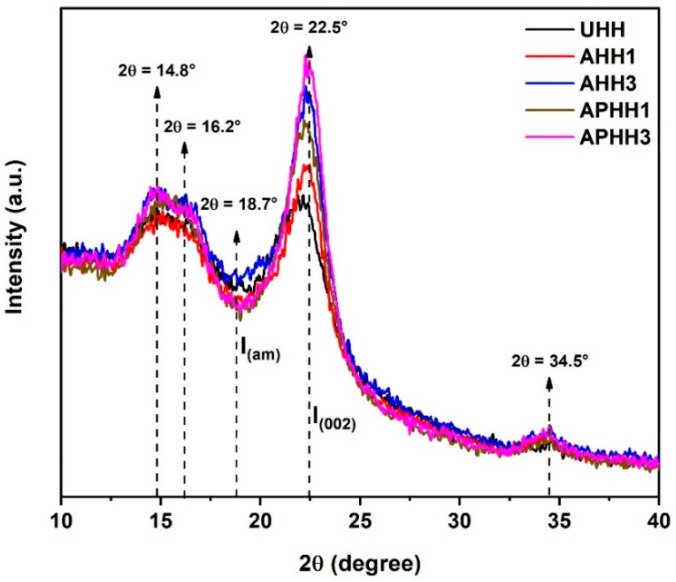
XRD patterns of untreated and treated hemp hurd powders.

The XRD patterns of untreated, alkaline, and alkaline/peroxide treated hemp hurd powders are presented in Figure 3. The characteristic peak of crystalline cellulose is located at 2θ = 22.5°; however, a small shift toward a higher diffraction angle was observed in the case of treated hemp hurd powder as compared to untreated hemp hurd powder. This peak is largest in magnitude for hemp hurd powder treated with three cycles, indicating that these samples have a high fraction of cellulose with respect to other components. On the other hand, for untreated hemp hurd powder, this peak is smaller in magnitude and shifted to a slightly lower angle, indicating that these samples have a lower overall fraction of cellulose. For hemp hurd powder which has undergone a single-cycle treatment, this peak exhibits an intermediate height and angle, indicating that these samples have an intermediate fraction of cellulose. 

In terms of the type of treatment, hemp hurd powder treated with alkaline/peroxide solution exhibited peaks with larger magnitude at 2θ = 22.5° compared to the alkaline-treated hemp hurd powder, indicating that the combined treatment is more effective at removing non-cellulosic components. For all samples, other noticeable peaks are observed at 2θ angles around 14.8, 16.2, and 34.5° corresponding to the crystallographic planes (1 1 0), (1 1 0), and (0 0 4), respectively [15,47]. The value I_m_ was selected at the local minimum near to 18°. For untreated hemp hurd powder, this minimum was observed at 2θ = 18.7° and this feature was slightly shifted to higher or lower diffraction angles for treated hemp hurd powders. The crystallinity index of each type of hemp hurd powder was calculated using the Segal method, and the results are summarized in Table 2. The results revealed that the crystallinity index of hemp hurd powder increased after performing alkaline and alkaline/peroxide treatments, where increasing the number of treatments resulted in a higher crystallinity index for each type of treatment. Moreover, hemp hurd powder which was treated using three cycles of alkaline/peroxide treatment (APHH3) exhibited the highest crystallinity index (*I_c_* = 53.28%), corresponding to an approximately twofold increase in crystallinity with respect to untreated hemp hurd powder (UHH). The increased crystallinity index of treated hemp hurd powders can be attributed to the removal of amorphous content of hemp hurd powder including hemicellulose, lignin, and pectin during treatments, which facilitated the rearrangement of cellulose chains [48]. Similar results regarding the crystallinity index of single-cycle alkaline-treated natural fibers have already been reported in other studies [16,41,49]. For instance, Vijay et al. observed a similar trend in the crystallinity index of alkaline-treated fibers owing to the removal of amorphous constituents, resulting in relaxation of cellulose chains [41]. A.N. Balaji and K.J. Nagarajan evaluated the crystallinity index of treated and untreated Saharan aloe vera cactus leaf (SACL) fibers using the Segal method [16]. They observed that the intensity of the peak corresponding to the crystalline fraction of cellulose (*I*_002_) is increased in the XRD pattern of treated fibers, resulting in a higher crystallinity index as compared to untreated fibers. They attributed this observation to the removal of non-crystalline components such as hemicellulose, pectin, and wax.

### 3.4. Thermal Stability of Hemp Hurd Powders

**Figure 4:**
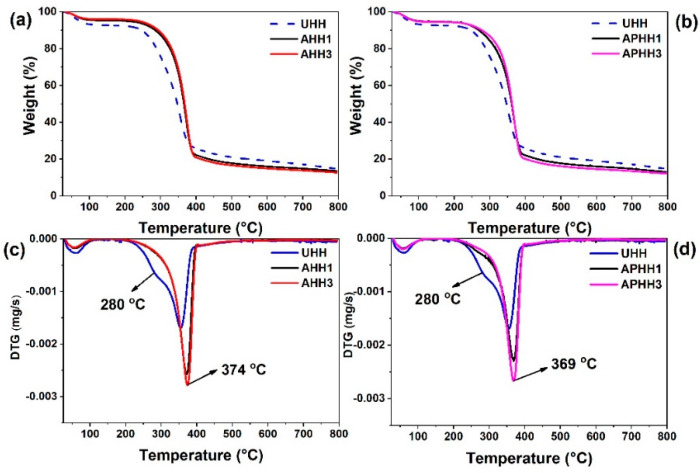
TGA thermograms of untreated, alkaline-treated (**a**), and alkaline/peroxide treated (**b**) hemp hurd powders, DTG thermograms corresponding to untreated, alkaline-treated (**c**), and alkaline/peroxide treated (**d**) hemp hurd powders.

TGA results of alkaline and alkaline/peroxide treated hemp hurd powders are shown in Figure 4a,b, respectively. *T*_10_, corresponding to the temperature at which 10% weight loss has occurred, and *T*_50_, the temperature at which 50% weight loss has occurred, were used as the reference parameters to evaluate the thermal stability of the hemp hurd powders. Extracted *T*_10_ and *T*_50_ values of untreated and treated hemp hurd powders are summarized in Table 3. It can be inferred from Table 3 that both *T*_10_ and *T*_50_ values increased for treated hemp hurd powders, indicating that the thermal stability of the treated hemp hurd powders increased in comparison to the untreated hemp hurd powder. This might be due to the removal of thermally unstable contents of hemp hurd powder such as hemicellulose, pectin, and wax during the treatments [15,41,50]. Moreover, in both of the treatments, an increase in the number of treatments further increased the *T*_10_ values of the hemp hurd powders, indicating continued removal of hemicellulose and pectin during the successive treatments. 

The derivative of thermogravimetric (DTG) thermograms of alkaline and alkaline/peroxide treated hemp hurd powders are presented in Figure 4c,d, respectively. For all samples, the first decomposition peak was detected between ambient temperature and 115 °C, which is associated with the removal of water [16]. This peak was observed for all of the hemp hurd powders: however, untreated hemp hurd powder (UHH) experienced a higher mass loss in this range than treated hemp hurd powders, revealing the fact that more water was absorbed by UHH due to their higher hydrophilicity. For UHH, a second decomposition peak was observed at 280 °C, which could be linked to the degradation of hemicellulose and partly lignin, which is in accordance with the results reported by Kabir et al. [51]. This peak was not observed for treated hemp hurd powders, reflecting that the hemicellulose was effectively removed during alkaline and alkaline/peroxide treatments. The main decomposition peak of both untreated and treated hemp hurd powders occurred in the range from 320–400 °C, which accounts for the decomposition of cellulose content in the hemp hurds. Maximum mass loss of UHH was observed at 356 °C, while this peak shifted to 374 and 369 °C for AHH3 and APHH3 samples, respectively. This observation indicates that the components with lower thermal stability were removed during three cycles of each treatment type.

### 3.5. Thermal Analysis of Biocomposites

**Figure 5:**
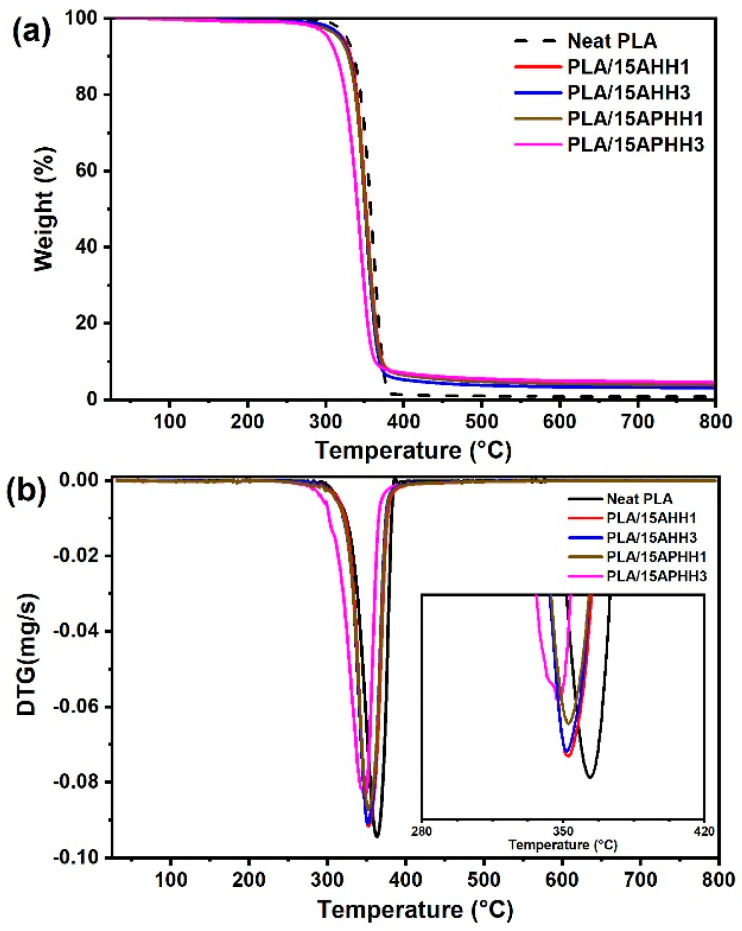
TGA (**a**) and DTG (**b**) thermograms of PLA and its biocomposites for 15 wt. % filler loading.

TGA and DTG thermograms of neat PLA and PLA/hemp hurd powder biocomposites containing 15 wt. % of hemp hurd powder are presented in Figure 5a,b. Figure 5a shows that all samples (neat PLA and PLA/hemp hurd powder biocomposites) exhibited a single-step degradation process. The initial decomposition temperature (*T_i_*, corresponding to the temperature at which the sample mass has decreased by 5%), final decomposition temperature (*T_f_*, corresponding to the temperature at which the sample mass has decreased by 90%), the temperature of maximum mass loss rate (*T_peak_*, corresponding to the temperature of the peak in DTG thermogram), and the percentage of char residue for each sample were determined from TGA and DTG thermograms and are represented in Table 4. It was found that the addition of hemp hurd powder resulted in a reduction in both initial and final decomposition temperatures of the biocomposites as compared with neat PLA. This observation can be attributed to the fact that the thermal stability of both hemicellulose and cellulose is lower than the thermal stability of PLA [52]. Similar results have been reported in the literature in the case of natural fiber reinforced PLA biocomposites [18,52]. Among the biocomposites, PLA/15AHH1 and PLA/15AHH3 exhibited *T_i_* of 322 and 320.4 °C, respectively; indicating better thermal stability in terms of initial decomposition temperature compared to PLA/15APHH1 and PLA/15APHH3 samples, with *T_i_* values of 317.3 and 303.5 °C, respectively. However, in terms of the final decomposition temperature, PLA/15APHH1 exhibited the highest *T_f_* value of all biocomposites (373.6 °C). The PLA/15APHH3 biocomposite was found to be the least thermally stable sample, as it exhibited the lowest initial and final decomposition temperatures. This observation could result from excessive delignification during the three cycles of successive alkaline/peroxide treatment process, which may have degraded the structure of the hemp hurds [53].

Char residue (residual mass) at 799 °C for all of the biocomposites was found to be higher than that of PLA, as presented in Table 4. This observation could be directly linked to the presence of hemp hurd powder in the PLA matrix due to the fact that—unlike PLA—lignocellulosic fibers have a charring step in their thermal degradation process, where at the temperatures higher than lignin’s degradation temperature range (200–500 °C) [54], both lignin and cellulose lead to the formation of char [55].

TGA and DTG curves corresponding to the composites containing 5 and 15 wt. % of treated hemp hurd powders are shown in the Supplementary Information, Figure S4. The PLA/5AHH3 and PLA/15AHH1 samples exhibited better thermal stability considering the initial decomposition temperature with *T_i_* values of 327 and 322 °C, respectively. PLA/5APHH3 and PLA/15APHH1 yielded higher final degradation temperatures (*T_f_*) of 376.1 and 373.6 °C, respectively. For all biocomposites, increasing the filler content resulted in a higher char residue at 799 °C. This char corresponds to the constituents of the hemp hurd powder (cellulose and lignin), which typically leave residue even at these high temperatures [56]. These observations are in accordance with the previously reported results by Singh et al., who observed that increasing cellulose fiber content in PLA-based biocomposites resulted in higher char residue at 600 °C [7].

To ensure the processibility of PLA/hemp hurd powder biocomposites using melt-processing techniques, the thermal stability of hemp hurd powder within the processing temperature range is required. Considering the initial degradation temperature of biocomposites containing 15 wt. % of treated hurd powder exceeds 250 °C, it can be inferred that melt processing of PLA/hemp hurd powder biocomposites at the melting temperature range of PLA (130–180 °C) [2] would not result in thermal degradation of the hemp hurd powder.

**Figure 6:**
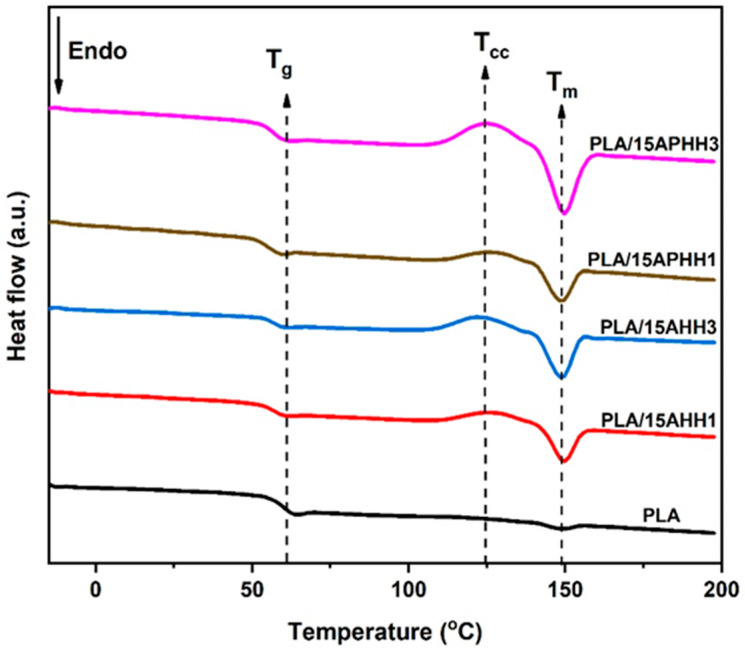
DSC thermograms of PLA and its biocomposites for 15 wt. % filler loading.

DSC thermograms corresponding to the second heating cycle of PLA and its biocomposites containing 15 wt. % of hemp hurd powder are given in Figure 6, and the relevant data were extracted and are summarized in Table 5. It was found that incorporation of hemp hurd powder lowered the glass transition temperatures (*T_g_*) of the biocomposites. This reduction was most prominent for the samples containing hemp hurd powder that underwent a single cycle of treatment (PLA/15APHH1 and PLA/15AHH1).

Each of the thermograms corresponding to the biocomposites exhibited peaks corresponding to a cold crystallization temperature (*T_cc_*), and a melting temperature (*T_m_*). However, a *T_cc_* peak was not present in the thermogram of PLA, reflecting the amorphous nature of this material. Similarly, neat PLA exhibited only a small melting peak at around 149.1 °C. For each of the biocomposites, only very small variations in melting temperatures were observed (ranging from 148.8 to 149.7 °C). Biocomposites containing three-cycle treated hemp hurd powder showed lower cold crystallization temperatures compared to the ones comprised of single-cycle treated hemp hurd powder. A reduction in cold crystallization temperature was observed previously for PLA upon the addition of increasing concentrations of pulp fibers, which were reasoned to act as heterogeneous nucleation sites [57]. Here, the results indicate that the heterogeneous nucleation of PLA occurred preferentially on hemp hurd powder that was exposed to a higher number of chemical treatment cycles.

PLA (without hemp hurd powder) exhibited the lowest crystallinity (1.3%), while the addition of treated hemp hurd powder resulted in a higher crystallinity. Biocomposites containing three-cycle treated hemp hurd powder yielded the highest crystallinity values of 18.7% and 18.5%, corresponding to PLA/15APHH3 and PLA/15AHH3, respectively. The increase in crystallinity of samples that occurred due to the incorporation of hemp hurd powder supports the idea that the treated hemp hurd powder acts as a nucleating agent in the PLA matrix, leading to the alignment of the PLA chains [3,58]. DSC results of biocomposites for various filler proportions (5, 10, 15 wt. %) are presented in the Supplementary Information, Figure S5, for further clarification of the impact of filler loading on thermal properties of the biocomposites. Figure S5 shows that increasing filler loading led to a further decrease in the glass transition temperature (*T_g_*) of the biocomposites, where the lowest *T_g_* was observed for the biocomposites with 15 wt. % filler content. It was also observed that increasing filler loading increased the crystallinity of the biocomposites.

### 3.6. Mechanical Properties of Biocomposites

**Figure 7:**
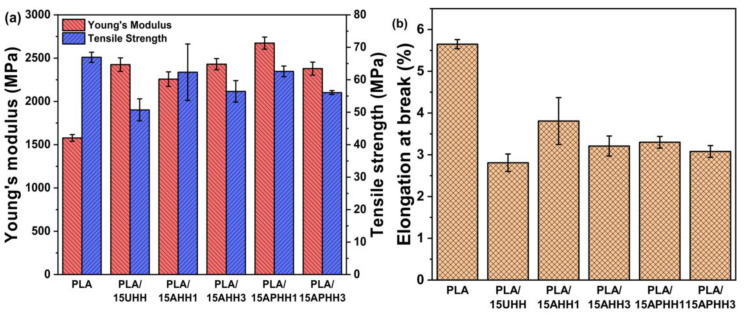
Mechanical properties of PLA and PLA/hemp hurd powder biocomposites with 15 wt. % filler loading. (**a**) Young’s modulus and tensile strength, (**b**) elongation at break (note: average values and standard deviation of five independent measurements are shown).

Tensile properties of PLA and its biocomposites containing various proportions (5, 10, and 15 wt. %) of untreated and treated hemp hurd powder were evaluated. To better understand the impact of hemp hurd powder on mechanical properties of biocomposites, samples were prepared with 15 wt. % filler loading of untreated and treated hemp hurd powders, and the Young’s modulus, tensile strength, and elongation at break of the samples are presented in Figure 7. Tensile results indicated that the addition of both untreated and treated hemp hurd powders to the PLA matrix resulted in a significant increase in Young’s modulus, which is likely related to both the higher stiffness of hemp hurds and the increase in crystallinity of the material [59].

The addition of 15 wt. % hemp hurd powder (prepared using the various treatment methods) resulted in a decrease in both elongation at break and tensile strength. The measured value of elongation at break for neat PLA was 5.7%, which was decreased to the range of 3.8~2.8% upon the addition of 15 wt. % of hemp hurd powders. In the case of tensile strength, the value for neat PLA was determined to be 66.9 MPa. For the PLA/15UHH sample, the obtained value of tensile strength was 50.7 MPa, corresponding to a 24% reduction in comparison to neat PLA. The reduction in tensile strength exhibited by biocomposites formed with untreated hemp hurd powder (UHH) with respect to PLA can be ascribed to inferior filler-matrix interaction caused by the high hydrophilicity of UHH. This powder contained hydrophilic components such as hemicellulose and lignin, which are incompatible with the hydrophobic PLA matrix.

Performing chemical treatments on the hemp hurd powder improved the filler-matrix compatibility to some extent, although the tensile strength values of the biocomposites were all lower than those of neat PLA. Nonetheless, each of the biocomposites with treated hemp hurd powders showed higher tensile strength values compared to the PLA/15UHH sample. The chemical treatments increased the surface roughness of hemp hurd powder (as shown in SEM results), which is expected to improve the filler-matrix interfacial interaction. This can be achieved by enhancing mechanical interlocking mechanism between filler and polymer matrix [60], caused by the higher level of surface roughness, which can lead to higher friction once the filler is pulled out of the matrix. This mechanism can eventually promote the mechanical properties of the biocomposites reinforced with treated hemp hurd powders [46]. Among the biocomposites containing treated hurd powders, PLA/15APHH1 yielded the highest tensile strength with a value of 62.6 MPa, showing a 6.5% decrease compared to neat PLA and a 23% increase in comparison to the sample containing untreated hemp hurd powder (PLA/15UHH). The efficient removal of hemicellulose, lignin, pectin, and wax from hemp hurd powder during alkaline/peroxide treatment led to an enhanced filler-matrix interaction in the case of the PLA/APHH1 biocomposite.

In addition to exhibiting the highest tensile strength of the biocomposites, PLA/15APHH1 was determined to have the highest Young’s modulus (2673.6 MPa), indicating an increase of 70% over neat PLA and a 10% increase as compared to PLA/15UHH. The tensile properties of the fabricated PLA/hemp hurd powder biocomposites are in agreement with the literature. B.A. Khan et al. evaluated the mechanical properties of PLA/hemp hurd biocomposites for various proportions of untreated hemp hurds (10, 20, and 30 wt. %) [61]. They found that incorporation of hemp hurds decreased the tensile strength of the biocomposites in comparison to neat PLA, whereas increasing the filler content further decreased the tensile strength of the biocomposites. They attributed this observation to the inadequate fiber wetting which led to inferior fiber-matrix interaction [61]. Johari et al. also reported similar results regarding Young’s modulus and strength in the case of PLA/cellulose fiber biocomposites [58]. They observed that the addition of cellulose fibers to PLA resulted in a decrease in tensile strength and ductility of fabricated biocomposites, while increasing the Young’s modulus of biocomposites.

The tensile strength values of PLA/15AHH1 and PLA/15APHH1 samples were found to be comparable. However, it was noted that the incorporation of three-cycle treated hemp hurd powder yielded lower tensile strength for the same filler loading in either case of alkaline and alkaline/peroxide-treated hemp hurd powder. This observation can be attributed to the excessive delignification and degradation of cellulose during the third cycle of treatments, resulting in poor mechanical properties as stated in the work of Malenab et al., who observed that increasing the immersion time of abaca fibers in NaOH solution resulted in lower tensile strength of fibers [53,62]. Here, for 15 wt. % filler content, biocomposites containing any kind of treated hemp hurd powders, regardless of the number of treatments, have been shown to have higher tensile strength than the PLA/15UHH biocomposite, indicating the impact of treatments on enhancing the mechanical properties—especially tensile strength—of the PLA/hemp hurd powder biocomposites.

**Figure 8:**
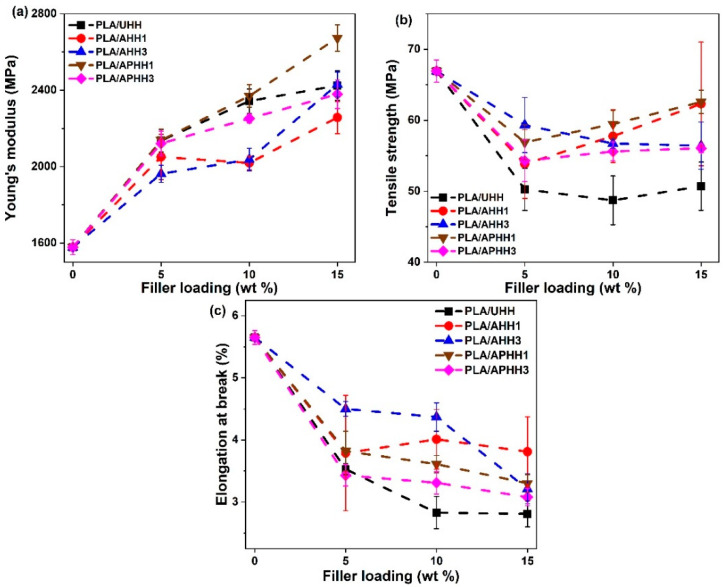
Young’s modulus (**a**), tensile strength (**b**), and elongation at break (**c**) of PLA biocomposites for 5, 10, and 15 wt. % filler loading (note: average values and standard deviation of five independent measurements are shown; lines are drawn solely in the interest of clarity).

The effect of filler loading on tensile properties of the biocomposites is shown in Figure 8, which shows the mechanical properties of biocomposites containing 0, 5, 10, and 15 wt. % hemp hurd powder in PLA for each treatment type (untreated, alkaline-treated, and alkaline/peroxide-treated). Figure 8a shows that the addition of both untreated and treated hemp hurd powders improved the Young’s modulus, where increasing the filler content in most of the cases led to higher Young’s modulus in biocomposites, indicating the stiffening effect of hemp hurds. Moreover, PLA/APHH1 was the only biocomposite containing treated hemp hurd powder that yielded a higher Young’s modulus than PLA/UHH in all of the filler contents stated in this study. In the case of the tensile strength (Figure 8b), it was found that the addition of 5 wt. % or more of any type of hemp hurd powder to PLA reduced the tensile strength of the biocomposites.

Figure 8c shows the elongation at break for composites containing varying concentrations of hemp hurd powder in a PLA matrix. The addition of any concentration of hemp hurd powder—treated or untreated—resulted in a reduction in elongation at break compared with neat PLA. This observation is directly associated with the stiffening effect of the hemp hurd powder in the PLA matrix, which results in relatively brittle biocomposites as compared to neat PLA. It can also be inferred that increasing the filler content further reduced the flexibility of the biocomposites in most of the samples. At higher filler contents (10 and 15 wt. %), biocomposites formed with untreated hemp hurd powder showed the lowest elongation at break values, likely due to inferior filler-matrix interaction resulting in improper stress transfer between the matrix and filler. In contrast, each of the biocomposites prepared with treated hemp hurd powder, regardless of the number of treatments, showed higher elongation at break values at 10 and 15 wt. % filler loading as compared to the biocomposites formed with untreated hemp hurd powder (although these values were still lower than those of neat PLA). This observation highlights the impact of chemical treatments in enhancing the filler-matrix interaction and hence the stress transfer between the two.

## 4. Conclusions

The aim of this study was to determine whether hemp hurd powder could be used as a low-cost filler (at 15 wt. %) in PLA without compromising the overall properties of the polymer. It was found that when untreated hemp hurd powder (ground to less than 500 μm) was blended with PLA, the tensile strength of the resulting composite decreased. By utilizing one or three cycles of alkaline and alkaline/peroxide treatments on hemp hurd powder before compounding with PLA, a higher tensile strength was achieved than for composites prepared using untreated hemp hurd powder. Of all of the biocomposites, the one containing 15 wt. % hemp hurd powder treated with a single cycle of alkaline/peroxide treatment (PLA/15APHH1) exhibited the best mechanical results in terms of tensile strength and Young’s modulus, exhibiting a 23% increase in tensile strength and 10% increase in Young’s modulus in comparison to the samples containing 15 wt. % untreated hemp hurd powder, and a 6.5% decrease in tensile strength and 70% increase in Young’s modulus as compared to the neat PLA. One drawback of the biocomposites is that the elongation at break—which is already relatively low for neat PLA—is further reduced through the addition of each type of the filler, particularly the untreated hemp hurd powder. DSC results indicated that treated hemp hurd powder could act as a nucleating agent leading to increased crystallinity of the PLA/hemp hurd powder biocomposites compared to PLA. Thermogravimetric analysis revealed that biocomposites containing treated hemp hurd powder exhibited proper thermal stability in the processing temperature range of PLA (130–180 °C) indicating the fact that this range of temperature does not pose any detrimental risk to the thermal stability of treated hemp hurds, and that these materials could subsequently be processed using thermal techniques such as injection molding, compression molding, and 3D printing without degrading the biocomposites.

Overall, these results suggest that treated hemp hurd powder can be used as a low-cost additive to reduce the amount of PLA required without compromising the overall properties of the material. Additional studies on both degradability and total cost of the composites (including materials and processing costs) would be beneficial towards establishing the applicability of these materials in applications such as packaging. Moreover, further studies on the barrier properties, such as water vapor transmission rate and oxygen transmission rate of the prepared biocomposites, are required to evaluate their potential to be used in food packaging applications.

## Figures and Tables

**Table 1:** Designated names for hemp hurd powders and prepared biocomposites.

Biocomposites	Filler Loading (wt. %)	Filler Type	Type of Treatment	Number of Cycles of Treatment
PLA/5UHH	5	UHH	Untreated	-
PLA/5AHH1	5	AHH1	Alkaline	1
PLA/5AHH3	5	AHH3	Alkaline	3
PLA/5APHH1	5	APHH1	Alkaline/peroxide	1
PLA/5APHH3	5	APHH3	Alkaline/peroxide	3
PLA/10UHH	10	UHH	Untreated	-
PLA/10AHH1	10	AHH1	Alkaline	1
PLA/10AHH3	10	AHH3	Alkaline	3
PLA/10APHH1	10	APHH1	Alkaline/peroxide	1
PLA/10APHH3	10	APHH3	Alkaline/peroxide	3
PLA/15UHH	15	UHH	Untreated	-
PLA/15AHH1	15	AHH1	Alkaline	1
PLA/15AHH3	15	AHH3	Alkaline	3
PLA/15APHH1	15	APHH1	Alkaline/peroxide	1
PLA/15APHH3	15	APHH3	Alkaline/peroxide	3

**Table 2:** The crystallinity index of untreated and treated hemp hurd powders.

Sample	UHH	AHH1	AHH3	APHH1	APHH3
Crystallinity index (%)	27.1	40.2	42.6	39.7	53.3

**Table 3:** *T*_10_ and *T*_50_ values of hemp hurd powders extracted from TGA curves.

Sample	*T*_10_ (°C)	*T*_50_ (°C)
UHH	246.5	347.1
AHH1	284.8	365.6
AHH3	294.0	368.4
APHH1	269.4	361.8
APHH3	278.6	363.3

**Table 4:** Data obtained from TGA results of PLA and its biocomposites.

Sample	*T_i_* (°C)	*T_f_* (°C)	*T_peak_* (°C) at DTG	Char Residue (%) at 799 °C
PLA	327.0	378.5	363.4	0.8
PLA/15AHH1	322.0	373.0	353.1	3.9
PLA/15AHH3	320.4	372.2	352.7	3.2
PLA/15APHH1	317.3	373.6	353.1	3.8
PLA/15APHH3	303.5	365.8	347.3	4.6

**Table 5:** DSC data corresponding to PLA and its biocomposites.

Sample	*T_g_* (°C)	*T_cc_* (°C)	*T_m_* (°C)	Δ*H_m_* (J/g)	*X_C_* (%)
PLA	60.4	-	149.1	1.28	1.3
PLA/15AHH1	56.1	125.3	149.6	10.62	13.0
PLA/15AHH3	56.4	122.3	148.9	15.13	18.5
PLA/15APHH1	55.0	125.7	148.8	9.11	11.2
PLA/15APHH3	56.5	124.8	149.7	15.28	18.7

## Data Availability

Not applicable.

## Supplementary Materials

The following are available online at https://www.mdpi.com/article/10.3390/polym13213786/s1, Figure S1: Received hemp hurds (a), untreated hemp hurd powder (UHH) (b), treated hemp hurd powder including AHH1(c), AHH3 (d), APHH1 (e), and APHH3 (f), Figure S2: The schematic of dumbbell-shaped specimen for tensile test, Figure S3: Tensile testing specimens after test; PLA (a), PLA/5UHH (b), PLA/10UHH (c), PLA/15UHH (d), PLA/5AHH1 (e), PLA/10AHH1 (f), PLA/15AHH1 (g), PLA/5AHH3 (h), PLA/10AHH3 (i), PLA/15AHH3 (j), PLA/5APHH1 (k), PLA/10APHH1 (l), PLA/15APHH1 (m), PLA/5APHH3 (n), PLA/10APHH3 (o), and PLA/15APHH3 (p), Figure S4: TGA thermograms of PLA and its biocomposites for 5 wt. % (a) and 15 wt. % (b) filler loading; DTG thermograms corresponding to PLA and its biocomposites for 5 wt. % (c) and 15 wt. % (d) filler content, Figure S5: DSC thermograms of PLA and its biocomposites for (a) 5 wt. %, (b) 10 wt. %, and (c) 15 wt. % filler content.
