# Supplementary material for: Valorization of Hemp Hurds as Bio-Sourced Additives in PLA-Based Biocomposites
Source: Polymers (Basel). 2021 Nov 1;13(21):3786. doi: 10.3390/polym13213786 (PMC8587083; doi:10.3390/polym13213786)
Supplement: Supplementary file 1 [file polymers-13-03786-s001.zip › polymers-1424897-supplementary.pdf]

polymers

Supplementary materials for

Valorization of hemp hurds as bio-sourced additives in PLA-based biocomposites

Sina Momeni [1], Muhammad Safder [2], Mohammad Abu Hasan Khondoker [1,2], and Anastasia Elias [1,*]

[1] Donadeo Innovation Centre for Engineering, Department of Chemical and Materials Engineering, University of Alberta, Edmonton, AB T6G 1H9, Canada; smomeni@ualberta.ca (S.M.); safder@ualberta.ca (M.S.)
[2] Industrial Systems Engineering, Faculty of Engineering and Applied Science, University of Regina, Regina, SK S4S 0A2, Canada; Mohammad.Khondoker@uregina.ca (M.A.H.K.)
* Correspondence: aelias@ualberta.ca

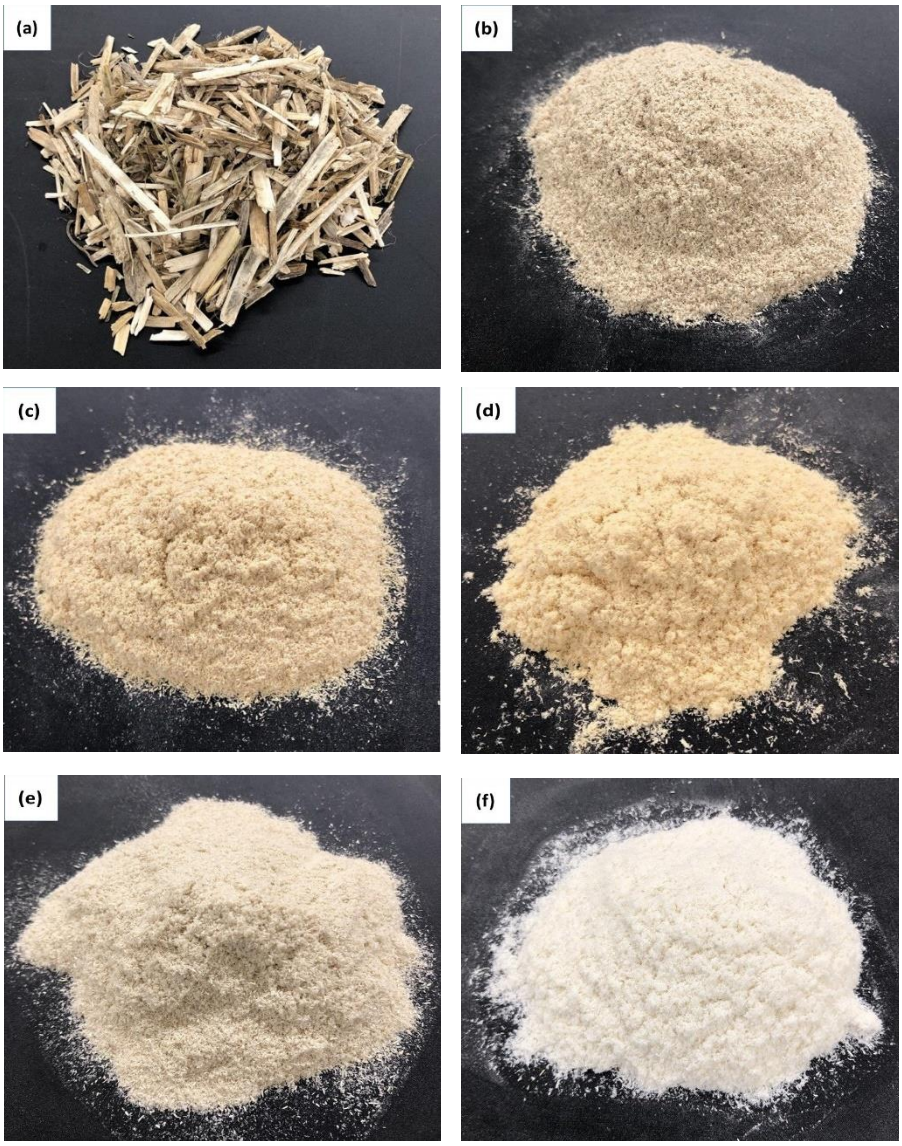

Figure S1. Received hemp hurds (a), untreated hemp hurds powder (UHH) (b), treated hemp hurds powders including AHH1(c), AHH3 (d), APHH1 (e), and APHH3 (f)

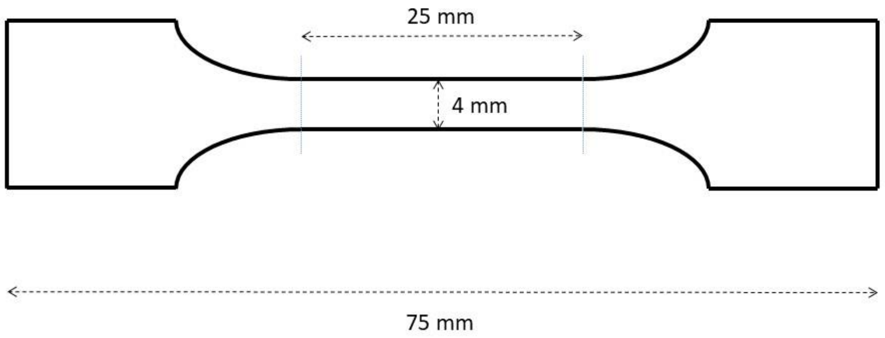

Figure S2. The schematic of dumbbell-shaped specimen for tensile test

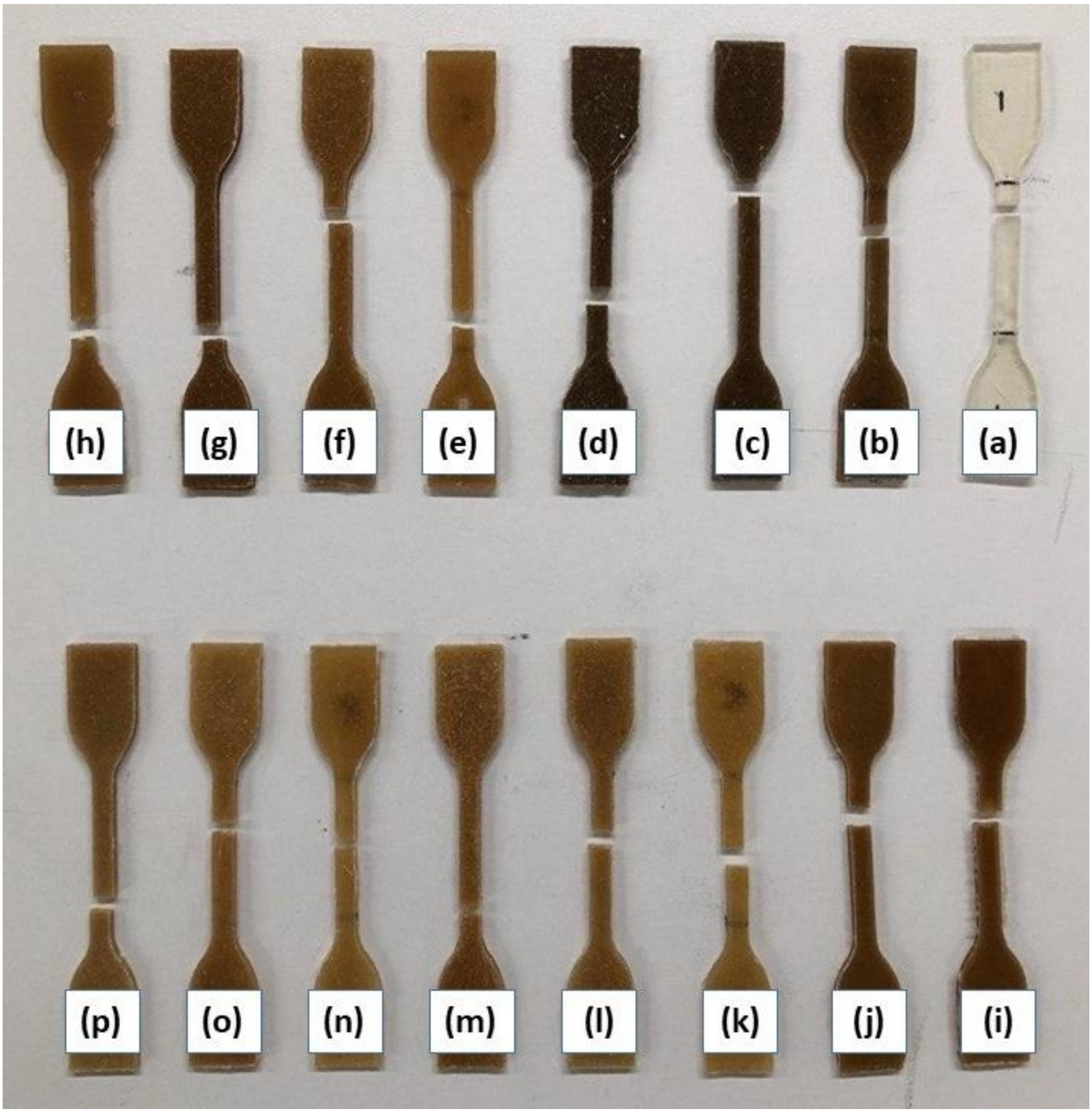

Figure S3. Tensile testing specimens after test; PLA (a), PLA/5UHH (b), PLA/10UHH (c), PLA/15UHH (d), PLA/5AHH1 (e), PLA/10AHH1 (f), PLA/15AHH1 (g), PLA/5AHH3 (h), PLA/10AHH3 (i), PLA/15AHH3 (j), PLA/5APHH1 (k), PLA/10APHH1 (l), PLA/15APHH1 (m), PLA/5APHH3 (n), PLA/10APHH3 (o), and PLA/15APHH3 (p)

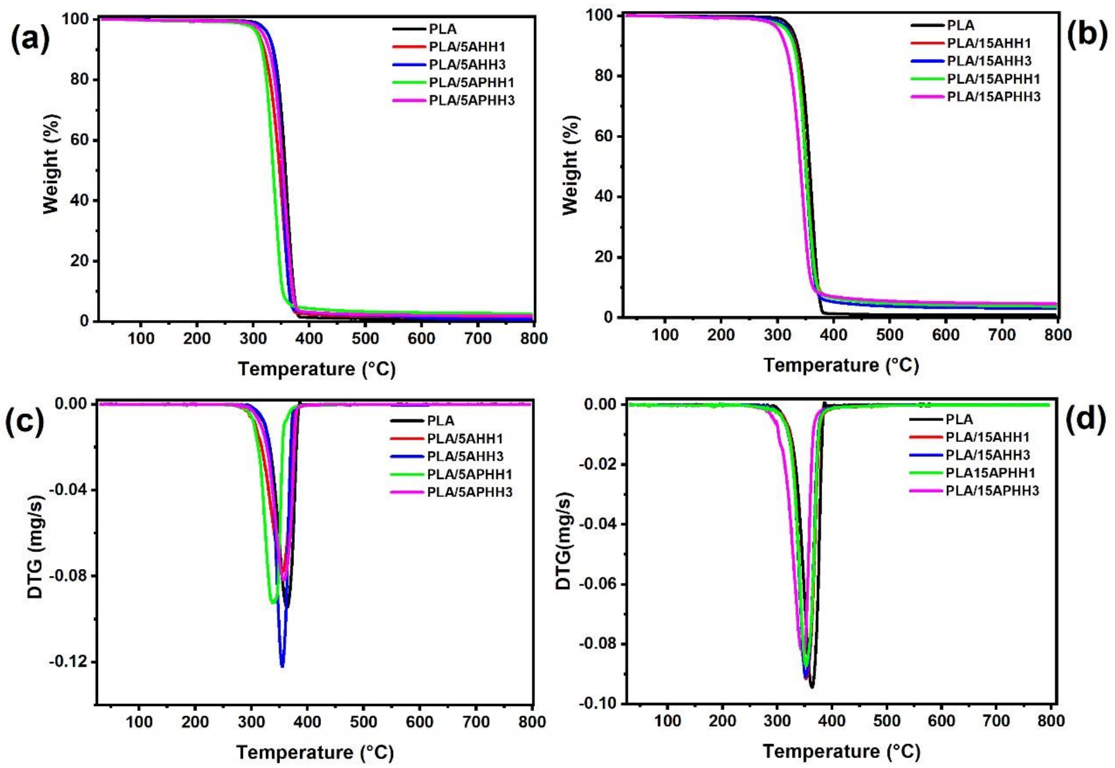

Figure S4. TGA thermograms of PLA and its biocomposites for 5 wt % (a) and 15 wt % (b) filler loading; DTG thermograms corresponding to PLA and its biocomposites for 5 wt % (c) and 15 wt % (d) filler content

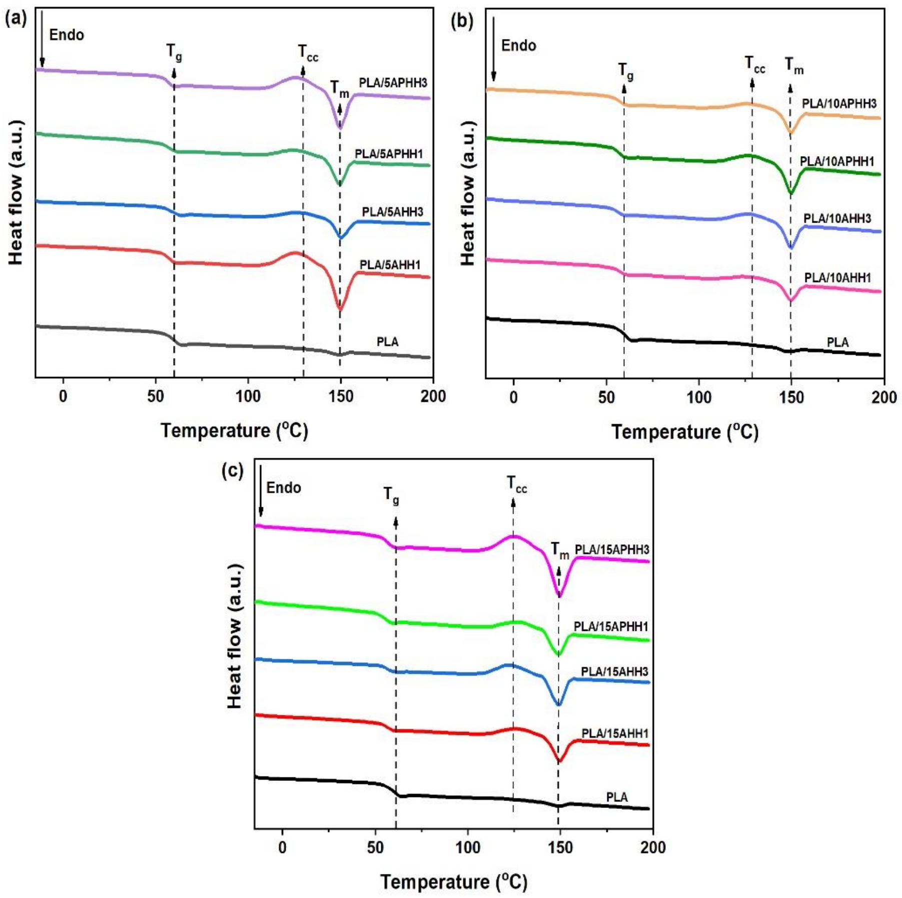

Figure S5. DSC thermograms of PLA and its biocomposites for (a) 5 wt %, (b) 10 wt %, and (c) 15 wt % filler content
